# Harnessing p97/VCP: A Transformative AAA+ ATPase Target for Next-Generation Cancer Therapeutics

**DOI:** 10.3390/cancers17182945

**Published:** 2025-09-09

**Authors:** Maria Janina Carrera Espinoza, Sarah K. Tucker, Sruthi Sureshkumar, Madison E. Gamble, Natalie L. Hakim, Sofia Orrantia, Claudia M. Espitia, Alexis B. Cruickshank-Taylor, Wei Wang, Kevin R. Kelly, Jennifer S. Carew, Steffan T. Nawrocki

**Affiliations:** 1 Department of Medicine, University of Arizona Cancer Center, Tucson, AZ 85724, USAsarahkt2015@gmail.com (S.K.T.);; 2 Department of Pharmacology and Toxicology, University of Arizona, Tucson, AZ 85724, USAwwang@pharmacy.arizona.edu (W.W.); 3 Bio5 Institute, University of Arizona, Tucson, AZ 85719, USA; 4 Division of Hematology, USC Norris Comprehensive Cancer Center, Pasadena, CA 91208, USA

**Keywords:** valosin-containing protein, VCP, p97, CB-5083, CB-5339, endoplasmic reticulum stress, cancer

## Abstract

Increased basal protein synthesis activity is a hallmark feature that distinguishes many types of malignant cells from their normal counterparts. The survival and proliferation of cancer cells are tightly linked to functional unfolded protein response (UPR) and endoplasmic reticulum (ER)-associated degradation (ERAD) pathways due to their high rates of protein synthesis. The evolutionarily conserved AAA+ ATPase valosin-containing protein (VCP)/p97 facilitates the extraction of proteins from organelles, chromatin, and protein complexes to target them for ubiquitin–proteasome system (UPS)-mediated degradation. p97 plays a key role in protein quality control and in the maintenance of protein homeostasis through its regulation of ERAD. The disruption of p97 activity leads to an accumulation of undegraded proteins, triggers the UPR, and can culminate in proteotoxic cell death. Given this, p97 inhibition offers an opportunity to selectively kill cancer cells that exhibit high basal protein synthesis rates. This review explores p97’s molecular structure, diverse cellular roles, and clinical potential with a particular focus on CB-5083 and CB-5339, the only p97 inhibitors to date that have advanced into clinical trials. We discuss their mechanisms of action, clinical trial outcomes, and the transformative potential of rational combination strategies to maximize their therapeutic potential. By integrating foundational biological insights with translational perspectives, we highlight p97 as a precision target for cancer treatment.

## Simple Summary

Cancer cells produce large amounts of proteins to support their rapid growth, which makes them vulnerable to disruptions in protein disposal systems. One key protein called valosin-containing protein (VCP) or p97 helps remove damaged or excess proteins and is often overactive in tumors. This review explains how p97 supports cancer cell survival and why blocking its activity may be an effective way to treat cancer. We highlight how experimental drugs that inhibit p97 have shown promise in preclinical studies and early clinical trials and we discuss how combining p97 inhibitors with other treatments could improve outcomes. By better understanding the role of p97 in cancer, researchers and clinicians may be able to develop new therapies that target this important cellular process more precisely and effectively.

## 1. Introduction

Protein homeostasis is critical for cell survival and is achieved through a tightly controlled balance between protein synthesis and degradation. Two major mechanisms govern protein turnover. The ubiquitin–proteasome system (UPS) is responsible for the elimination of the majority of proteins, whereas autophagy mediates the degradation of organelles and selected proteins with long half-lives [1,2]. Malignant cells typically exhibit significantly elevated rates of protein synthesis compared with their normal counterparts [3]. This hallmark characteristic drives their proliferation and survival and creates an increased dependency on the cellular degradation machinery. Specific regulators of this process are frequently upregulated in cancer cells to manage the proteotoxic stress that results from their aberrant protein synthesis activity. This phenomenon provided the rationale to develop inhibitors of regulators of protein turnover for cancer therapy. The successful development of the proteasome inhibitor bortezomib established proof of concept that this approach may result in therapeutic benefit. Although proteasome inhibitors have demonstrated significant efficacy for the treatment of patients with multiple myeloma and mantle cell lymphoma, resistance to treatment is frequent and their effectiveness in treating solid tumors is limited [4]. Beyond that, the global inhibition of the proteasome produces unwanted side effects that hinder its clinical activity and tolerability. This prompted an intensive effort to develop new targeted inhibitors of more specific regulators of protein turnover with the goal of achieving a superior therapeutic index.

**Figure 1:**
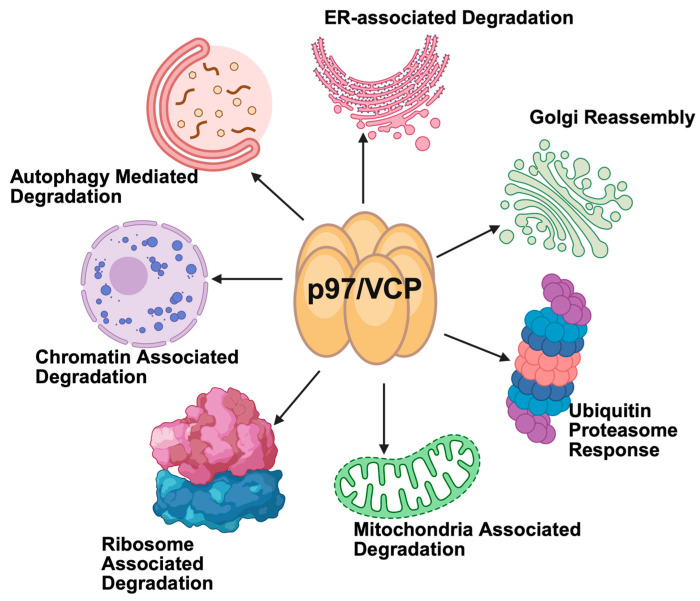
Multifaceted roles of p97 in cellular protein quality control. p97 participates in endoplasmic reticulum-associated degradation (ERAD) and additional key proteolytic pathways, including mitochondrial-associated degradation and ribosome-associated quality control to maintain protein homeostasis and regulate diverse cellular processes.

The valosin-containing protein (VCP)/p97 is a highly conserved member of the AAA+ family of ATPases. p97 is a hexameric protein that can interact with a wide number of protein cofactors through its N-terminal and C-terminal domains [5]. It was originally identified as a protein involved in the degradation of misfolded or damaged proteins in the endoplasmic reticulum (ER)-associated protein degradation (ERAD) pathway and is primarily localized in the cytosol and organelle membranes including the ER, Golgi, mitochondria, and endosomes. p97 also plays important roles in a multitude of other cellular processes such as membrane trafficking, chromatin remodeling, cell cycle regulation, DNA damage repair, endosomal sorting, lysosomal vesicle fusion, and autophagy (Figure 1). Given these essential functions, it is not surprising that p97 has been reported to be upregulated in a variety of tumor types including colorectal cancer, pancreatic cancer, non-small cell lung cancer, and hematological malignancies [6]. Its overexpression has also been linked to poor prognostic features including invasive phenotype, drug resistance, and disease recurrence [5,6,7,8].

Multiple studies have shown that the disruption of p97’s function promotes an accumulation of undegraded proteins, activation of the unfolded protein response (UPR), and induction of apoptosis [6,9,10]. Since cancer cells frequently overexpress p97, its inhibition may offer a targeted strategy to selectively kill cancer cells. This ignited a major effort to develop small molecule-targeted p97 inhibitors for cancer therapy.

To date, several p97 inhibitors have been developed, including compounds that bind in ATP-competitive and non-competitive manners that achieve reversible or irreversible inhibition [9,11,12,13]. CB-5083, the trailblazing first-in-class p97 inhibitor, forged a path from compelling preclinical efficacy to Phase I clinical trials and established proof-of-concept that targeting p97 has clinical potential [9,14]. CB-5339, its optimized successor, exhibits enhanced potency and has also advanced into clinical trials [15,16]. In this review, we explore p97’s functional role in malignant pathogenesis, the efficacy and mechanism of action of targeted p97 inhibitors, and the exciting possibilities of rational combination approaches to maximize therapeutic benefit. By blending a core biological understanding with practical treatment insights, we emphasize p97’s role as a key target for precision cancer therapy.

## 2. Structural Organization and Functional Domains of p97

**Figure 2:**
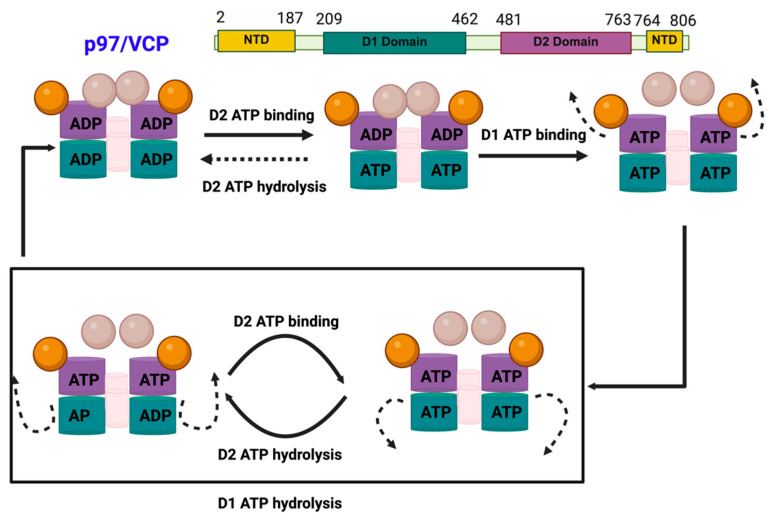
Structural organization and functional domains of p97.

p97 has four structural domains: a conserved N-terminal domain, two AAA ATPase domains (D1 and D2), and a C-terminal tail, all of which are essential for its function (Figure 2) [17,18,19,20,21]. The D1 and D2 domains are stacked in a head-to-tail manner and connect with a short polypeptide linker. The D1 domain primarily facilitates hexamer formation, whereas the D2 domain performs most of the ATPase activity [22]. The N-terminal domain is connected to the D1 domain by another short linker and is important for substrate recognition and its interaction with other cofactors [23]. The C-terminal tail of p97 has been reported to be involved in nuclear localization through interactions with other proteins [24,25]. Fundamentally, p97 functions as an interaction hub and at least 30 different cofactors have been shown to modulate p97-mediated processes [26,27,28,29].

## 3. p97’s Role in the Ubiquitin–Proteasome System (UPS)

The UPS is the principal cellular machinery responsible for the selective degradation of damaged, misfolded, or short-lived proteins. It plays a fundamental role in maintaining protein homeostasis, regulating cell cycle progression, orchestrating DNA repair, and modulating a wide range of intracellular signaling pathways [30]. The covalent attachment of polyubiquitin chains to target proteins by E1 ubiquitin-activating enzymes, E2 conjugating enzymes, and E3 ubiquitin ligases is central to this process. These polyubiquitinated substrates are then recognized and degraded by the 26S proteasome.

p97 acts as a segregase within the UPS [26,31]. It couples the energy of ATP hydrolysis to the extraction of ubiquitinated substrates from cellular compartments that are otherwise inaccessible to the proteasome. While p97 does not catalyze ubiquitination itself, it collaborates with a host of E3 ligases, adaptor proteins, and deubiquitinases to facilitate the delivery of substrates to the proteasome. This is particularly critical for membrane-anchored or complex-bound proteins, such as those embedded in the ER, chromatin, or cytoskeletal scaffolds, where direct access by the proteasome is sterically hindered.

A classic example of p97’s function within the UPS is its role in ER-associated degradation (ERAD), where it dislocates misfolded or unassembled proteins from the ER membrane for subsequent proteasomal degradation [32]. This extraction process is mediated by cofactors such as Npl4-Ufd1 and often coordinated with ER-resident E3 ligases such as Hrd1 and gp78. Beyond ERAD, p97 is also indispensable for other UPS-dependent processes including the turnover of stalled or damaged DNA replication complexes, disassembly of post-mitotic protein assemblies, unfolding of soluble proteins that cannot be unfolded by the proteasome, and clearance of defective ribosomal products [33,34,35]. p97 also contributes to the co-translational degradation of nascent polypeptides [36]. By extracting ubiquitinated, misfolded translation products from ribosomes and membrane translocons, p97 ensures that defective proteins are promptly cleared. This is a key mechanism of preserving proteome integrity through the prevention of aggregation-prone protein accumulation that can impair cellular function or lead to proteotoxic stress.

Through these multifaceted activities, p97 acts as a core orchestrator of protein quality control within the UPS. The dysregulation of p97-mediated UPS processes is implicated in various diseases, including neurodegenerative disorders and cancer. In neurodegenerative disorders, impaired UPS function can lead to protein aggregation, contributing to Alzheimer’s and Parkinson’s [37]. In cancer, disrupted UPS activity may exacerbate proteotoxic stress or alter signaling pathways, promoting tumor progression or resistance to therapies [38]. The critical role of p97 in maintaining UPS efficiency underscores its potential as a therapeutic target for addressing these disease states.

## 4. Regulation of p97 Activity

p97’s activity is tightly regulated by a combination of post-translational modifications, interacting proteins, and cellular stress responses. Collectively, these processes modulate its conformation and substrate binding capacity to ensure precise cellular function.

### 4.1. Post-Translational Modifications

Post-translational modifications (PTMs) are key regulators of p97 function and serve to modulate its conformation, cofactor binding, substrate interactions, and enzymatic activity [39]. Among these modifications, phosphorylation plays a particularly prominent role in fine-tuning p97’s activity in response to cellular cues. Phosphorylation can occur at multiple residues. However, one of the most functionally significant sites is the C-terminal serine 784 [40]. The phosphorylation of p97 at Ser784 alters the interaction landscape of p97 by modulating its affinity for specific adaptor proteins and substrates. This modification directly impacts the recruitment of cofactor complexes, thereby influencing p97’s ability to process ubiquitinated substrates in a context-dependent manner. Ser784 phosphorylation has been shown to be upregulated in response to cellular stress and is frequently overexpressed in aggressive cancer subtypes [41]. For example, in triple-negative breast cancer, elevated phosphor-Ser784 correlates with poor clinical outcomes [40]. Functionally, this site-specific phosphorylation enhances p97’s involvement in ERAD, DNA damage repair, and cell cycle progression. These findings underscore the importance of phosphorylation as a dynamic regulatory mechanism that expands the versatility of p97 and enables it to meet the evolving demands of diverse cellular environments.

Ubiquitination is another essential mechanism of p97 regulation that significantly influences its function across multiple cellular pathways [42]. p97 itself can be ubiquitinated by various E3 ubiquitin ligases, including Ufd2 and gp78, which modulate its activity, stability, and interactions with cofactors [43]. The U-box-type E3 ligase Ufd2 has been shown to polyubiquitinate p97, which enhances its recruitment to specific substrates during protein quality control processes. Similarly, the ubiquitination of p97 by gp78 (also known as AMFR), a membrane-associated RING-type E3 ligase involved in ER-associated degradation (ERAD), promotes retrotranslocation and substrate processing within the endoplasmic reticulum [44]. p97 undergoes dynamic ubiquitination and deubiquitination cycles, which facilitate the extraction of misfolded proteins from the ER membrane during ERAD [26]. Notably, the site-specific ubiquitination of p97 can influence its conformational states and modulate its ATPase activity. This suggests a feedback mechanism in which p97’s enzymatic function is coupled to its post-translational modification status. These findings underscore the sophisticated regulatory network governing p97 function and highlight ubiquitination as a critical determinant of its cellular specificity.

### 4.2. Cofactor Interactions

Cofactor interactions are central to the functional versatility of p97. They enable it to engage in a wide range of cellular processes by guiding its substrate specificity, subcellular localization, and mechanochemical activity [45]. p97 operates as a hub for diverse signaling pathways. This adaptability is largely conferred by its ability to bind a broad array of cofactors. Among the best-characterized are Npl4 (nuclear protein localization 4), Ufd1 (ubiquitin fusion degradation protein 1), and the adaptor protein p47 (NSFL1C) [46]. These cofactors associate primarily with the N-terminal domain of p97. However, additional regulation can occur through interactions with the C-terminal tail.

The Npl4-Ufd1 complex plays a fundamental role in mediating p97’s engagement with polyubiquitinated substrates in response to ERAD, DNA damage response, and chromatin-associated protein quality control [47]. Npl4 contains a zinc finger domain that binds ubiquitin chains. This enables the complex to bridge p97 to substrates marked for extraction and degradation. Ufd1 enhances this process by stabilizing the cofactor complex and assisting in substrate recruitment. Together, Npl4 and Ufd1 direct p97 to disassemble protein complexes or remove stalled factors from chromatin and membrane compartments.

In contrast, p47 modulates p97’s function in membrane trafficking, Golgi reassembly, and nuclear envelope formation [48]. Unlike Npl4-Ufd1, p47 does not rely on ubiquitin recognition. Rather, it directs p97 activity toward deubiquitinase recruitment and phospholipid-modifying enzymes. This facilitates the non-proteolytic remodeling of cellular structures. These distinct cofactor interactions not only determine the fate of p97-bound substrates, but also dictate the biological outcomes of its activity. This empowers the precise spatiotemporal regulation of protein homeostasis across different cellular compartments.

### 4.3. Cellular Stress Responses and Signaling Pathways

Cellular stress responses and signaling pathways add another layer of p97 regulation that enable it to respond to environmental and proteotoxic challenges. The UPR and heat shock response are key stress responses that impact p97 activity. The activation of the UPR modulates its function in response to protein misfolding or cellular stress [49]. This provides a mechanism for p97 to restore cellular homeostasis under proteotoxic conditions.

The activity of p97 is tightly regulated by several upstream signaling pathways that modulate its ATPase function, substrate processing, and interactions with cofactors. For example, Polo-like kinase 1 (PLK1) phosphorylates p97 at Thr76 [50]. This modification enhances p97’s chromatin-associated segregase activity. Oxidative stress also alters p97 activity by oxidizing key cysteine residues, most notably Cys522, within the D2 ATPase domain [51]. This affects its ATPase activity and substrate processing capacity. Furthermore, SUMOylation has been identified as a stress-responsive post-translational modification of p97 that alters its localization and function [52]. However, some disease-associated p97 mutations such as those that cause inclusion body myopathy with Paget’s disease of bone and frontotemporal dementia appear to impair this regulatory mechanism [53].

In the DNA damage response, ATM and ATR kinases indirectly regulate p97 recruitment and function by phosphorylating chromatin-bound cofactors and adaptors such as SPRTN [54]. This facilitates p97’s localization to stalled replication forks and double-strand breaks. Recent evidence suggests that ULK1/ULK2 kinases can regulate p97 function in stress granule clearance via phosphorylation [55]. This links p97 to stress-induced autophagy-related signaling. Finally, the AMPK family member Salt-Inducible Kinase 2 (SIK2) interacts with and phosphorylates p97 to stimulate its ATPase activity [56]. These collective mechanisms ensure that p97 activity is dynamically adapted to ensure the precise regulation of protein quality control, DNA repair, and stress adaptation.

Collectively, these signaling-driven stress responses ensure that p97’s activity is tightly controlled to meet the fluctuating demands of the cell under different conditions. The dysregulation of these mechanisms due to mutation, oncogenic stress, or chronic inflammation can lead to impaired protein quality control, and genomic instability contributes to cancer development and progression (Table 1).

## 5. ER-Associated Degradation (ERAD)

ER-associated degradation (ERAD) is a cellular process responsible for recognizing and disposing of misfolded or unassembled proteins in the ER to maintain protein homeostasis and prevent cellular dysfunction. p97 plays a central role in ERAD by extracting ubiquitinated substrates from the ER membrane and facilitating their delivery to the proteasome for degradation [9]. This process is essential for protein quality control and its dysregulation is linked to cancer and neurological disorders.

### 5.1. The ERAD Process

ERAD involves a coordinated sequence of steps to recognize, ubiquitinate, extract, and degrade misfolded proteins. In the ER lumen, misfolded or unassembled proteins are identified during protein folding and marked for degradation by the addition of ubiquitin moieties [60,61]. This recognition and ubiquitination prevent the accumulation of defective proteins and ensure that they are targeted for disposal. The ubiquitinated substrates are then translocated from the ER membrane to the cytosol where p97 facilitates their extraction. The ATPase activity of p97 is vital for dislocating these substrates and unfolding them to enable their translocation to the proteasome for degradation into peptides [62]. This multi-step process maintains cellular protein homeostasis by ensuring the efficient removal of defective proteins. The dysregulation of p97-mediated ERAD has been connected to cancer and other diseases.

### 5.2. The Central Role of p97 in ERAD

p97 functions as a key player in ERAD by orchestrating the extraction of ubiquitinated substrates from the ER membrane. Its role involves both mechanical extraction and coordination with other cellular components, as highlighted by several studies [60,63]. p97’s ATPase activity drives the energy-intensive process of dislocating substrates to ensure that they are properly unfolded and prepared for proteasomal degradation. This activity is critical for ERAD’s efficiency as it prevents the buildup of misfolded proteins that could trigger ER stress. As discussed earlier, p97 interacts with various cofactors that enhance its ability to recognize and extract ubiquitinated proteins. Additionally, p97 collaborates with ubiquitin ligases and deubiquitinating enzymes to regulate the ubiquitination status of substrates and control ERAD activity [64]. These interactions facilitate appropriate protein breakdown to maintain the balance necessary for optimal cellular health.

## 6. Autophagy and p97-Mediated Aggrephagy

Autophagy is a highly conserved cellular process involved in the degradation and recycling of damaged or unwanted cellular components [65]. This process plays a crucial role in maintaining cellular homeostasis by eliminating misfolded proteins, damaged organelles, and protein aggregates. Autophagy-related (ATG) proteins and other key molecular players orchestrate the formation of double-membrane structures called autophagosomes, which engulf cellular cargo for degradation [66,67]. Following autophagosome formation, fusion with lysosomes enables the degradation of cargo. This mechanism of nutrient recycling plays a significant role in maintaining cellular function, particularly under stress conditions. Aberrations in autophagic degradation have been implicated in a range of diseases including cancer and neurodegenerative disorders [68,69]. This process involves a series of events (initiation, nucleation, elongation, lysosomal fusion, and degradation) that are regulated by varied cell signals and stress [70,71].

Aggrephagy is a specialized form of autophagy responsible for the clearance of protein aggregates that serves to maintain cellular homeostasis under stress conditions. p97 has emerged as a key regulator of this process. Recent studies demonstrate that p97 overexpression reduces the accumulation of ubiquitinated proteins in autophagy-deficient cells overexpressing p62 [72,73]. This suggests that p97 and p62 may compete for binding to ubiquitinated substrates. This competitive interaction positions p97 as a regulatory switch that influences the selection and clearance of aggregated proteins.

Notably, p97’s role in aggrephagy is further modulated by histone deacetylase 6 (HDAC6), which determines the specificity of aggregate degradation. In the presence of HDAC6, p97 promotes selective aggrephagy by targeting ubiquitinated protein aggregates for autophagic degradation. However, in the absence of HDAC6, p97 facilitates non-selective aggrephagy to enable the degradation of aggregates regardless of their ubiquitination status [74,75]. This functional shift highlights the ability of p97 to fine-tune autophagic responses based on cellular context and the availability of regulatory cofactors. Through its interactions with key autophagy adaptors and cofactors, p97 integrates protein quality control mechanisms with autophagic clearance, ensuring the efficient disposal of cytotoxic aggregates. This activity is particularly important in proteotoxic stress and contributes to broader cellular adaptation mechanisms [74,76].

The dysregulation of p97-regulated autophagy has been implicated in several pathological states, most prominently cancer and neurodegenerative diseases. In cancer, enhanced autophagy may support tumor cell survival by alleviating proteotoxic stress [2,77,78,79]. This is especially important for rapidly proliferating or aneuploid cells with high rates of protein turnover. Conversely, in neurodegenerative disorders such as Alzheimer’s and Parkinson’s disease, impaired aggrephagy contributes to the accumulation of insoluble protein aggregates [80]. This exacerbates neuronal dysfunction and cell death. These disease associations highlight the therapeutic relevance of targeting p97 in contexts where autophagic clearance mechanisms are either exploited or compromised. As such, the pharmacological modulation of the p97 function represents a promising strategy to restore proteostasis and mitigate the progression of aggregation-associated diseases.

## 7. p97 in DNA Damage Response

The DNA damage response (DDR) is an essential cellular mechanism that preserves genomic stability by detecting, signaling, and repairing DNA lesions. The disruption of DDR pathways can lead to mutations, chromosomal aberrations, and malignant transformation. p97 plays an essential role in supporting DDR processes through the extraction and turnover of ubiquitinated proteins from chromatin. It facilitates the resolution of DNA damage by removing ubiquitylated proteins from sites of DNA lesions [81]. This creates access for repair machinery and ensures the proper turnover of regulatory proteins. The regulation of 53BP1 recruitment to DNA double-strand breaks (DSBs) is a key example of p97’s role in this process. This is mediated by p97’s extraction of the polycomb group protein L3MBTL1, which binds methylated histones and can obstruct 53BP1 access. By displacing L3MBTL1 from chromatin, p97 enables 53BP1 to localize to DSBs and initiate non-homologous end joining or influence repair pathway choice. p97 is also involved in processing interstrand crosslinks (ICLs) [82]. Although p97 does not directly repair ICLs, it supports the removal of ubiquitinated proteins that accumulate at ICL sites, facilitating lesion unhooking and processing by downstream repair pathways.

Another major function of p97 in DDR is its role in the homologous recombination repair (HRR) of DSBs. Studies have shown that p97’s interaction with HRR-associated factors is essential for the timely disassembly of protein complexes at stalled replication forks. The loss or inhibition of p97 results in persistent DSBs, chromosomal breaks, and defective HRR [83]. This demonstrates that its segregase activity is required for the resolution of recombination intermediates and the maintenance of genome integrity. Given its central role in facilitating chromatin-associated protein turnover during DNA repair, inhibiting p97 may sensitize cancer cells to genotoxic agents or exploit synthetic lethal vulnerabilities in tumors with existing DDR defects.

## 8. Chromatin Remodeling and Transcriptional Regulation

Beyond its established roles in protein degradation and DNA damage response, p97 has emerged as a critical regulator of chromatin remodeling and transcriptional control. Chromatin remodeling involves dynamic alterations in nucleosome positioning and composition to regulate DNA accessibility for transcription, replication, and repair. p97 contributes to this process through chromatin-associated degradation (CAD) [84]. It acts as a segregase extracting ubiquitin- or SUMO-conjugated proteins from chromatin to modulate transcription, replication, and DNA repair. This function ensures the proper progression of key biological events such as cell cycle progression and gene expression [63]. p97 also modulates transcriptional responses by disassembling protein complexes or removing post-translationally modified regulators from chromatin [85]. For example, p97 functions to provide negative feedback regulation of the UPR by facilitating the degradation of the transcription factor ATF4 during ER stress. It also promotes the removal and degradation of DNA damage response proteins such as L3MBTL1 and XPC to support transcriptional reprogramming and the activation of DNA repair pathways following genotoxic stress. Another well-characterized example of CAD is the p97-mediated turnover of the replication licensing factor Cdt1. During the S-phase, the removal of Cdt1 from chromatin is an essential event that prevents re-replication. p97 ensures the timely extraction and degradation of Cdt1 in partnership with specific ubiquitin ligases and cofactors. This preserves replication fidelity and cell cycle integrity.

Given its essential functions in the maintenance of genomic integrity, it is not surprising that its disruption can have severe consequences. The impairment of p97 activity leads to the accumulation of ubiquitin-conjugated proteins on chromatin. This cellular state is known as protein-induced chromatin stress (PICHROS), which disrupts DNA replication, transcription, and repair by physically obstructing access to DNA [84]. This leads to replication stress, activation of checkpoint responses, and genomic instability. PICHROS underscores the essential role of p97 in maintaining chromatin fluidity and preventing genotoxic stress associated with unresolved chromatin-bound protein complexes.

## 9. p97 and Cancer

Cancer cells frequently exhibit high rates of protein synthesis. This leads to significant proteotoxic stress due to the accumulation of misfolded proteins and non-stoichiometric protein complexes, which is often exacerbated by aneuploidy [37]. p97 relieves this stress by facilitating the UPS-mediated degradation of misfolded proteins to ensure cellular homeostasis and support tumor cell survival. This positions p97 as a non-oncogene addiction therapeutic target. Notably, inactivating mutations in p97 have not been reported in malignancies [13]. This indicates that cancer cells rely on wild-type p97 to sustain their proliferative capacity. This dependency creates a therapeutic window as p97 inhibition can selectively induce proteotoxic crisis and apoptosis in cancer cells while sparing normal cells with lower protein synthesis demands.

Dysregulated p97 activity contributes to tumor survival, invasion, metastasis, and therapy resistance across multiple cancers [7]. In colorectal cancer, high p97 expression drives invasion and recurrence [86]. In pancreatic ductal adenocarcinoma (PDAC), elevated p97 levels are linked to reduced survival [59]. Similarly, in non-small cell lung cancer (NSCLC), p97 promotes metastatic potential and chemoresistance [57]. These associations underscore p97’s role in promoting tumor progression and highlight its potential as a biomarker for aggressive disease (Table 2).

The upregulation of p97 in cancers and their dependency on its function make it an attractive therapeutic target [93]. Consistent with this, inhibiting p97 disrupts protein homeostasis, triggers proteotoxic stress, and selectively induces tumor cell death. This approach is particularly effective against malignant cell types with high secretory phenotypes or protein synthesis demands [14,94,95,96]. The absence of somatic p97 mutations enhances its therapeutic appeal as inhibitors can target the wild-type protein.

## 10. p97 May Be a Transformative Therapeutic Target

**Figure 3:**
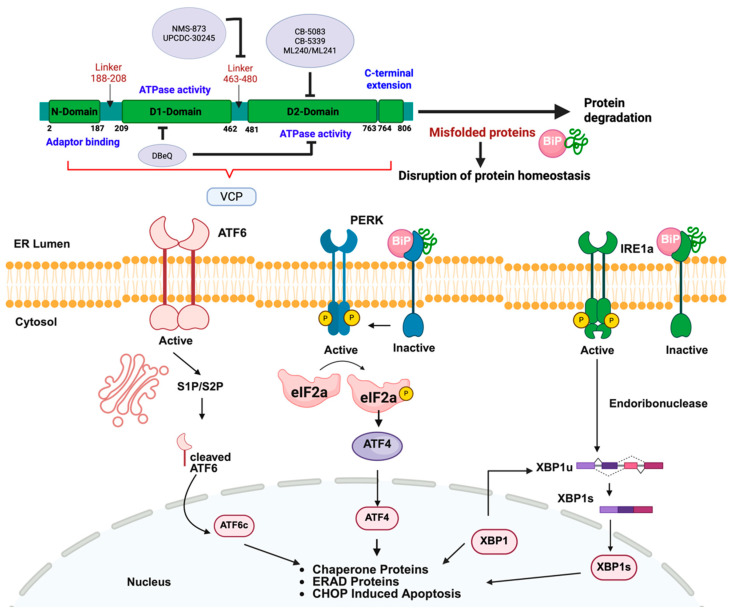
Mechanistic model of p97 inhibitors and endoplasmic recticular stress.

Given its central role in multiple cellular processes including protein homeostasis, chromatin remodeling, and the DNA damage response, p97 has emerged as a compelling therapeutic target. The dysregulation of p97 activity is implicated in a variety of diseases, including cancer, neurodegeneration, and viral infection [7,97]. Consequently, the development of small-molecule p97 inhibitors has become an active area of research. p97 inhibitors are designed to disrupt the ATPase activity or cofactor interactions of p97 in a manner that interferes with its roles in protein homeostasis and other cellular processes (Figure 3). Since many cancer cells are heavily reliant on the UPR and ERAD pathways due to high protein synthesis rates and possibly aneuploidy, the inhibition of p97 induces irresolvable proteotoxic stress-mediated cell death. Therefore, p97 is an actionable vulnerability in cancer cells, particularly in those that exhibit constitutive levels of ER stress, such as MM. It is also notable that p97 inhibitors have been reported to induce higher UPR activation than bortezomib, indicating that they may display differential anticancer efficacy compared to proteasome inhibitors [9]. Taken together, p97 inhibitors represent a promising class of anticancer agents with clinical potential that warrants further investigation.

These inhibitors are classified as ATP-competitive, which target the D1 or D2 ATPase domains to block ATP hydrolysis, or allosteric, which bind outside these domains to alter p97’s conformation. Reversible inhibitors allow transient inhibition, while irreversible inhibitors permanently disable p97. The different classes of p97 inhibitors are characterized by diverse therapeutic profiles, which may enable precision treatment (Table 3).

### 10.1. CB-5083 and CB-5339

CB-5083 is a first-in-class selective and orally bioavailable inhibitor of p97 and is also the first targeted p97 inhibitor to enter clinical trials. It is a reversible, ATP-competitive compound that specifically targets the critical substrate processing D2 ATPase domain of p97 [9,14]. Mechanistically, CB-5083 disrupts proteostasis by inhibiting p97’s ability to extract and process ubiquitinated proteins. This leads to activation of the UPR and subsequent apoptosis. Preclinical studies have demonstrated that CB-5083 has broad anti-tumor activity across a wide range of malignant cell types including multiple myeloma, colorectal, and pancreatic cancers [9,14,98]. Its anticancer mechanism of action is characterized by the potent induction of ER stress and apoptosis. Multiple studies have shown that the administration of CB-5083 to mice bearing cancer xenografts significantly antagonizes disease progression. Although early clinical trials of CB-5083 were halted due to off-target ocular toxicity, its development has paved the way for next-generation p97 inhibitors with improved safety profiles.

**Figure 4:**
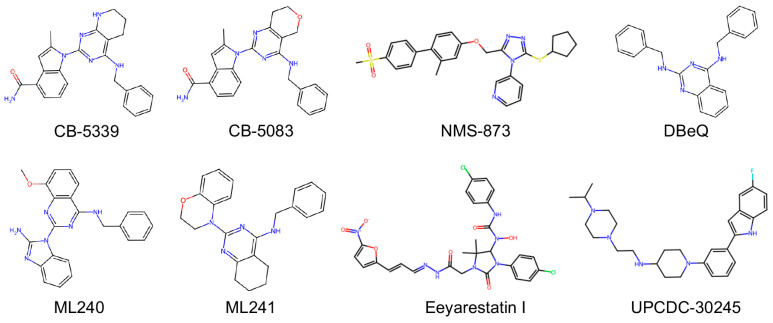
Chemical structures of p97 inhibitors.

CB-5339 is a second-generation orally bioavailable inhibitor of p97 that was developed to overcome the limitations of its predecessor, CB-5083, including off-target toxicities and suboptimal pharmacokinetics (Figure 4). Like CB-5083, CB-5339 also targets the D2 ATPase domain of p97. However, it has enhanced potency and improved pharmacological properties. Preclinical studies have demonstrated that CB-5339 effectively inhibits p97 function, triggers the accumulation of ubiquitinated proteins, and induces proteotoxic stress [15,16]. The compound’s improved oral bioavailability, target specificity, and tolerability profile support its advancement as a clinical candidate [13]. CB-5339 is currently under evaluation in early-phase clinical trials for hematologic malignancies and solid tumors, where it may be particularly effective in tumors reliant on p97 for proteostasis and survival under cellular stress.

### 10.2. N^2^,N^4^-Dibenzylquinazoline-2,4-diamine (DBeQ)

DBeQ was the first small-molecule inhibitor identified to selectively target p97 [11]. It is a reversible, ATP-competitive inhibitor and structural studies have confirmed that DBeQ exerts its inhibitory activity by specifically binding to the nucleotide-binding pocket of the D2 ATPase domain [99]. This competitive binding effectively prevents ATP hydrolysis. Unlike proteasome inhibitors, which target downstream components of the UPS, DBeQ interferes with p97’s upstream substrate-processing function and disrupts the extraction and turnover of ubiquitinated proteins from various cellular compartments. Functionally, DBeQ has been shown to block multiple p97-dependent pathways, including ubiquitin fusion degradation (UFD), ERAD, and autophagosome maturation [100]. These effects mirror phenotypes observed with RNAi-mediated p97 knockdown and validate the compound’s on-target activity. Despite its potency, DBeQ’s use in vivo has been limited due to suboptimal pharmacokinetic properties. However, it remains a valuable research tool that laid the groundwork for the development of more drug-like p97 inhibitors.

### 10.3. 2-(2-Amino-1H-benzo[62]imidazol-1-yl)-N-benzyl-8-methoxyquinazolin-4-amine (ML240) and 2-(2H-Benzo[62][1,4]oxazin-4(3H)-yl)-N-benzyl-5,6,7,8-tetrahydroquinazolin-4-amine (ML241)

ML240 and ML241 are two additional small molecule inhibitors developed to target the ATPase activity of p97. These compounds are also ATP-competitive and selectively bind to the D2 domain of p97. Both ML240 and ML241 effectively inhibit ERAD, but only ML240 has been shown to inhibit autophagy and induce apoptosis. In contrast, ML241 exhibits milder cellular effects and does not induce apoptosis to the same extent [12]. The differential activity between these two inhibitors may reflect the importance of dual pathway inhibition in triggering cell death. Specifically, the induction of apoptosis may require the simultaneous disruption of both autophagy and proteasome-dependent degradation, which is achieved more effectively by ML240 [12,101]. These findings suggest that the degree and specificity of p97 inhibition can result in distinct cellular outcomes and provide a rationale for further structure−activity relationship (SAR) optimization.

### 10.4. NMS-873

NMS-873 is regarded as one of the most potent and selective allosteric inhibitors of p97 identified to date. Unlike ATP-competitive inhibitors that target the nucleotide-binding sites directly, NMS-873 binds to a cryptic allosteric site within the D2 ATPase domain [102]. Its binding interferes with the inter-subunit communication required for coordinated ATP hydrolysis by disrupting interactions between the arginine finger of the NMS-873–bound subunit and the γ-phosphate of ATP in the adjacent subunit [102,103]. This alters p97 hexamer conformational dynamics without disrupting its oligomeric state and allows NMS-873 to stabilize an inactive conformation of the enzyme [104]. The high potency and selectivity of NMS-873 make it a valuable tool compound for dissecting p97 function and a promising lead for therapeutic development [105].

### 10.5. UPCDC30245

UPCDC-30245 is another allosteric inhibitor of p97. Studies demonstrate that UPCDC-30245 has broad and potent anti-proliferative effects against cancer cells with IC_50_ values in the nanomolar to low micromolar range [106]. In HCT116 colorectal cancer cells, UPCDC-30245 upregulates the transcription of the UPR genes *CHOP* and *ATF3*, while markedly increasing the key autophagy markers p62 and LC3-II, which suggests impaired autophagic flux. As demonstrated by Wang et al., UPCDC-30245 disrupts endo-lysosomal degradation by inhibiting early endosome formation and reducing lysosomal acidity [107,108]. This disruption contributes to its effects on autophagy and protein homeostasis. However, UPCDC-30245 exhibits weak effects on protein ubiquitination and overall UPR activation. This indicates a selective impact on p97-dependent pathways. These findings highlight UPCDC-30245’s potential as a tool for studying p97’s role in autophagy and lysosomal function as well as a potential therapeutic for cancers reliant on p97-mediated proteostasis.

### 10.6. Eeyarestatin 1

Eeyarestatin I (ESI) is another small molecule p97 inhibitor that disrupts ERAD [109]. ESI blocks the p97-dependent dislocation of misfolded proteins from the ER to the cytosol, which causes them to accumulate in the ER. This disruption induces ER stress, activates the UPR, and triggers apoptosis. Mechanistically, ESI interferes with p97-associated deubiquitinating enzymes. This antagonizes the deubiquitination of ERAD substrates and further inhibits their proteasomal degradation [110]. Its mechanism of action highlights the nuanced cellular consequences that can occur through differing modes of p97 inhibition.

## 11. Clinical Trials of p97 Inhibitors

### 11.1. CB-5083

As mentioned earlier, CB-5083 is a first-in-class clinical p97 inhibitor developed by Cleave Biosciences. The compound first advanced to first-in-human Phase I clinical trials in 2015. This marked a significant milestone in the validation of p97 as a therapeutic target. Two trials were initiated: NCT02243917 in patients with advanced solid tumors and NCT02223598 in those with relapsed/refractory multiple myeloma (MM) and lymphoid hematological malignancies. In NCT02243917, patients with various solid tumors including colorectal cancer and NSCLC received escalating oral doses of CB-5083. While no RECIST-defined partial responses were observed, 25% of patients achieved stable disease for 3 to 6 cycles. In the parallel trial (NCT02223598) focused on MM, the median progression-free survival (PFS) in this cohort was approximately 3 months, which fell short of the benchmarks set by proteasome inhibitors such as bortezomib. The clinical investigation of p97 inhibitors in patients with MM is based on their hypersensitivity to ER stress.

Pharmacodynamic data demonstrated robust target engagement. Within 24 h, peripheral blood mononuclear cells (PBMCs) exhibited a three-fold increase in polyubiquitinated proteins and a four-fold increase in CHOP expression. This is consistent with p97 inhibition and activation of the UPR. However, the trials also revealed significant limitations. Dose-limiting toxicities (DLTs) including nausea, fatigue, and grade 3 diarrhea prevented further dose escalation. Approximately 30% of patients discontinued treatment due to adverse effects. Plasma concentrations of CB-5083 reached ~1 μM, which aligned with preclinical efficacy thresholds. However, tumor biopsies revealed inconsistent drug penetration. This suggested potential pharmacokinetic barriers to achieving uniform target inhibition in vivo. CB-5083 also exhibited off-target effects on phosphodiesterase 6 (PDE6), which led to ophthalmological side effects such as transient night blindness.

Although the CB-5083 program did not proceed to Phase II trials and was discontinued by 2017, its clinical trajectory parallels the early development of proteasome inhibitors like bortezomib where initial setbacks eventually led to therapeutic breakthroughs. CB-5083’s clinical data established proof-of-concept for pharmacological p97 inhibition and paved the way for the development of next-generation inhibitors with improved potency, selectivity, and safety profiles.

### 11.2. CB-5339

CB-5339 is a second-generation orally bioavailable inhibitor of p97 that was developed by Cleave Therapeutics as a successor to CB-5083. CB-5339 also selectively targets the D2 ATPase domain of p97, but features enhanced metabolic stability, bioavailability, and reduced off-target effects on PDE6, which were key limitations that hindered CB-5083’s clinical progression. CB-5339 entered clinical evaluation in 2019 through two Phase I trials. NCT04402541 is a dose escalation study in patients with relapsed/refractory acute myeloid leukemia (AML) and intermediate/high-risk myelodysplastic syndromes (MDSs) [111]. NCT04372641 is a parallel trial in patients with advanced solid tumors and lymphomas. The clinical focus on AML is related to p97 being identified as a high vulnerability in preclinical studies following p97 chemical inhibition in AML models [16].

The hematologic malignancy study (NCT04402541) utilized a standard 3 + 3 dose-escalation design to evaluate safety, pharmacokinetics, and preliminary efficacy. CB-5339 was administered orally on a 4 days on, 3 days off schedule in 28-day cycles to allow recovery from potential proteostasis-related toxicities. CB-5339 was well tolerated up to the maximum dose level tested with manageable adverse events and no treatment-related deaths. Importantly, the ocular toxicity previously observed with CB-5083 was not reported following treatment with CB-5339. This reflects an improved safety profile. Pharmacodynamic analyses confirmed target engagement with increases in polyubiquitinated protein accumulation and upregulation of stress markers like CHOP in peripheral blood mononuclear cells. These findings mirrored the expected molecular response to p97 inhibition and were consistent across multiple dose levels. Preliminary efficacy signals were modest but encouraging. Several patients with AML or MDS achieved stable disease or reductions in blast counts. This supports continued investigation in this patient population.

The solid tumor/lymphoma trial (NCT04372641) followed a similar design for patients with advanced or relapsed/refractory malignancies. However, the study was withdrawn due to sponsor reprioritization. Casi Pharmaceuticals has acquired the global intellectual property rights of CB-5339 from Cleave Biosciences to continue its development. Taken together, the clinical trials of CB-5339 provide proof-of-concept for selective p97 inhibition with a significantly improved safety profile compared to the first-generation compound. Although objective responses have been limited thus far, the ability to achieve pharmacologically relevant drug levels with tolerable dosing schedules represents a meaningful advancement in the development of p97-targeted therapies (Table 4). Ongoing and future trials may focus on combination strategies to exploit potential synthetic lethalities and enhance therapeutic efficacy.

## 12. Rational Combination Approaches with p97 Inhibitors

### 12.1. Protein Homeostasis Disruptors/Inducers of ER Stress

Given p97’s central role in protein quality control, combining p97 inhibitors with other agents that disrupt proteostasis such as proteasome inhibitors or ER stress inducers offers a rational strategy to enhance therapeutic efficacy. While p97 inhibition primarily interferes with the extraction and processing of ubiquitinated substrates in pathways such as the UPS and ERAD, proteasome inhibitors block the final degradation of these substrates. When combined, these agents create a synergistic proteotoxic burden by impairing both upstream and downstream components of the degradation pathway [94].

Under normal physiological conditions, the accumulation of misfolded proteins in the ER activates the UPR, which is mediated through three principal stress sensors: Inositol-requiring enzyme 1α (IRE1α), PKR-like ER kinase (PERK), and activating transcription factor 6 (ATF6). These sensors coordinate transcriptional and translational responses that reduce protein synthesis, upregulate chaperones, and enhance the degradation of misfolded proteins [112]. When ER stress is prolonged or excessive, these pathways shift from adaptive to pro-apoptotic. This primarily occurs through the activation of CHOP and downstream caspases.

The dual inhibition of p97 and the proteasome or co-treatment with ER stress–inducing agents such as bortezomib, tunicamycin, or thapsigargin can overwhelm the cell’s ability to resolve misfolded proteins. This strategy enhances CHOP expression, polyubiquitinated protein accumulation, and caspase activation, resulting in greater tumor cell apoptosis. Moreover, pharmacologic or genetic interference with UPR mediators has been shown to sensitize cells to p97 inhibition by further amplifying ER stress and promoting apoptosis [113]. These findings support the notion that UPR signaling not only serves as a protective mechanism, but also represents a vulnerability in tumors with high secretory or proteostatic demand. Together, these insights underscore the therapeutic potential of combinatorial regimens that integrate p97 inhibition with proteasome inhibitors, ER stress inducers, or UPR pathway modulators. Such approaches may be particularly effective in malignancies characterized by heightened protein synthesis, dysregulated ER stress responses, or dependence on proteostasis for survival.

### 12.2. DNA Damage/PARP Inhibitors

p97 plays a critical function in the DDR by facilitating the extraction and turnover of ubiquitinated proteins from chromatin during DNA repair [114]. Inhibiting p97 impairs these processes, which leads to persistent DNA lesions, replication stress, and compromised genomic integrity. This makes p97 inhibition an attractive strategy for combination with DNA repair-targeted therapies.

Poly(ADP-ribose) polymerase-1 (PARP-1) is a key nuclear enzyme that acts as a sensor of DNA damage. Upon detecting DNA strand breaks, PARP-1 catalyzes the addition of poly(ADP-ribose) chains (PARylation) to itself and target proteins. This facilitates the recruitment of DNA repair complexes to the site of damage. In this role, PARP-1 supports multiple aspects of genome maintenance as it is involved in base excision repair (BER) by recognizing single-strand breaks (SSBs). p97 also contributes to replication fork stability including the regulation of fork elongation and recognition of unligated Okazaki fragments during lagging strand synthesis [112]. Additionally, PARP-1 promotes DNA repair or the initiation of programmed cell death via DNA fragmentation when the damage level exceeds repair capacities. Finally, PARP-1 also coordinates with scaffold proteins such as XRCC1 and other core repair factors at DNA damage sites to accelerate repair processes and preserve genomic stability [115,116,117].

p97 inhibitors can also prolong PARP1 trapping to enhance PARP inhibitor-induced cytotoxicity in homologous recombination-defective tumor cells [118]. Preclinical studies have shown that combining p97 inhibitors with PARP inhibitors leads to enhanced accumulation of γH2AX (a marker of DNA double-strand breaks), increased CHOP expression, and activation of apoptotic pathways. These findings suggest a promising strategy for selectively targeting cancer cells while sparing normal tissues with intact DNA repair mechanisms. Together, these data support the further development of p97 and PARP inhibitor combinations as a rational therapeutic approach in cancers with high replicative stress, defective DDR pathways, or increased dependence on proteostasis for survival.

### 12.3. Signaling Pathway Inhibitors

Combining p97 inhibitors with antagonists of key signaling pathways represents another rational and promising strategy for cancer therapy that leverages the functional crosstalk between protein homeostasis and oncogenic signaling networks. This dual-targeted approach aims to exploit cancer-specific vulnerabilities by disrupting critical survival and proliferation mechanisms simultaneously. p97 inhibition compromises protein degradation, DNA repair, and stress response pathways, which are functions that are often co-opted or upregulated in malignant cells. When combined with signaling pathway inhibitors such as those targeting PI3K/AKT/mTOR, MAPK/ERK, or NF-κB, this strategy can amplify cellular stress and tip the balance toward apoptosis [119,120]. For example, p97 inhibition may impair the degradation of oncogenic signaling intermediates and consequently sensitize cells to kinase blockade. Conversely, the inhibition of survival pathways may reduce the cellular capacity to buffer the proteotoxic stress induced by p97 inhibitors.

This combination approach offers several therapeutic advantages. First, it promotes enhanced cytotoxicity through the synergistic induction of apoptosis by capitalizing on the interplay between signaling networks and stress response mechanisms. Second, it improves efficacy by overcoming intrinsic or acquired resistance to monotherapies. Third, it provides broader pathway coverage by targeting multiple cancer hallmarks simultaneously. Finally, it offers the potential for a lower dosing of each agent, which may reduce toxicity.

### 12.4. Autophagy Inhibitors

Autophagy inhibitors like hydroxychloroquine (HCQ) represent another promising class of agents to combine with p97 inhibitors [121,122]. This approach is particularly attractive for cancers that rely heavily on autophagy and aggrephagy for their survival under proteotoxic stress. While p97 drives the extraction and degradation of ubiquitinated protein aggregates through the UPS and aggrephagy, autophagy serves as a compensatory mechanism to clear damaged organelles and misfolded proteins that accumulate in stressed cells. Blocking both pathways simultaneously may overwhelm the cellular proteostasis machinery and trigger apoptosis.

HCQ inhibits autophagy by disrupting lysosomal acidification and blocking autophagosome–lysosome fusion, which impairs the terminal degradation of autophagic cargo. When used in combination with p97 inhibitors, HCQ may effectively prevent the clearance of p97 inhibitor-stimulated aggregates, leading to a significant increase in p62-positive aggregates and defective autophagic clearance [123]. The high accumulation of undegraded aggregates that results from the inability of cancer cells to compensate for the simultaneous inhibition of both the UPS and autophagy pathways may induce synergistic levels of cell death.

This combination strategy specifically targets p97’s role in aggrephagy. Tumors characterized by high levels of proteotoxic stress, such as those with aneuploidy, rapid proliferation, or high metabolic activity, are particularly dependent on aggrephagy for survival [3]. As such, the dual inhibition of p97 and autophagy may be especially effective in these contexts and offer a strategy to induce synthetic lethality. Ongoing preclinical studies and early-phase clinical trials may help define the optimal contexts and dosing strategies for such combination therapies.

### 12.5. Histone Deacetylase Inhibitors

Histone deacetylase (HDAC) inhibitors are a mechanistically rational class of agents to combine with p97 inhibitors due to their ability to modulate chromatin accessibility, protein turnover, and cellular stress responses [124]. By modulating both epigenetic regulation and proteostasis, this combination strategy targets cancer cells that rely heavily on dynamic chromatin remodeling and protein quality control systems [125]. HDAC inhibitors such as vorinostat promote histone acetylation, chromatin relaxation, and increased genomic accessibility to transcriptional and DNA repair machinery. When combined with p97 inhibitors, which disrupt CAD, this increased chromatin accessibility facilitates the accumulation of ubiquitinated chromatin-bound proteins that are normally extracted by p97.

HDAC6 plays a distinct role in maintaining proteostasis by regulating aggresome formation and the autophagic clearance of misfolded proteins [126,127]. In addition to pan-HDAC inhibitors, the selective inhibition of HDAC6 has also shown strong synergy with p97 inhibitors. In mantle cell lymphoma models, the selective HDAC6 inhibitor ACY-1215 (ricolinostat) enhanced the cytotoxic effects of the p97 inhibitor CB-5083 [75]. This therapeutic combination led to marked increases in caspase activation and apoptosis and the downregulation of DNA repair proteins such as BRCA1 and 53BP1. This dual inhibition approach also led to increased levels of DNA damage, impaired DSB repair, diminished tumor growth, and extended overall survival in xenograft models. Collectively, these data highlight the therapeutic potential of combining p97 inhibitors with HDAC inhibitors, whether broad-spectrum or HDAC6-selective, to exploit cancer cell dependence on both chromatin plasticity and proteostasis. Further investigation in preclinical models and early-phase clinical trials is warranted to evaluate the safety, optimal scheduling, and mechanistic biomarkers predictive of response in these dual-targeting strategies.

### 12.6. Immunotherapies

The integration of p97 inhibition with the immune checkpoint blockade represents a novel and emerging strategy in cancer treatment [128]. While p97 is best known for its roles in proteostasis, chromatin regulation, and DNA repair, recent studies have revealed its additional involvement in immune regulation, including the modulation of major histocompatibility complex class I (MHC-I) antigen presentation. Given the importance of MHC-I expression for the effective recognition of tumor cells by cytotoxic T lymphocytes, strategies that augment this pathway may enhance the efficacy of immunotherapeutic agents [129].

p97 contributes to the processing and degradation of misfolded or defective proteins via the UPS and ERAD pathways, which intersect with the antigen processing machinery. The inhibition of p97 may increase the intracellular pool of peptides available for MHC-I loading by impairing degradation and enhancing the accumulation of antigenic peptide precursors. This could lead to the elevated surface expression of MHC-I molecules and improved tumor immunogenicity. Combining p97 inhibitors with immune checkpoint inhibitors like anti-PD-1 or anti-CTLA-4 antibodies could therefore result in a more immunostimulatory tumor microenvironment [130]. By simultaneously increasing antigen presentation and relieving T cell exhaustion, this dual approach holds the potential to elicit a more robust and sustained anti-tumor immune response. Additionally, p97 inhibition could alter the tumor cytokine milieu and influence immune cell infiltration. This strategy remains in the early stages of investigation. Additional preclinical studies are needed to define the immunomodulatory effects of p97 inhibition, identify optimal combinations, and determine the tumor types most likely to benefit. Nonetheless, these preliminary findings suggest that targeting p97 may not only disrupt tumor-intrinsic survival pathways but also enhance tumor immune visibility. This provides a clear rationale for combining p97 inhibitors with immunotherapies in future clinical trials.

## 13. Future Directions

p97 has emerged as a multifunctional regulator of proteostasis, chromatin dynamics, and stress responses. These hallmarks are increasingly exploited by cancer cells to sustain survival under stress conditions. Acting at the crossroads of critical cellular processes such as ERAD, the UPS, CAD, aggrephagy, DNA damage repair, and transcriptional regulation, p97 enables tumor cells to navigate both proteotoxic and genotoxic stress. Notably, its consistent overexpression across malignancies and the absence of inactivating mutations underscore p97’s role as a non-oncogene addiction. These factors make it a highly compelling therapeutic target in cancer.

The development of small molecule p97 inhibitors has validated this concept in preclinical models with compounds like CB-5083 and CB-5339, demonstrating the capacity to disrupt multiple survival pathways and induce tumor regression. Although the clinical translation of p97 inhibitors has faced significant challenges, including dose-limiting toxicities, CB-5339 has shown promising signs of efficacy in patients with hematologic malignancies and solid tumors in early-phase clinical trials. The collective clinical experience to date with p97 inhibitors highlights the need for refined therapeutic strategies to fully harness p97’s vulnerability.

Recent advances in the structural and biochemical characterization of p97, coupled with innovative discovery methods, have revealed numerous allosteric and regulatory sites. These discoveries offer the potential to develop next-generation inhibitors with improved selectivity and safety profiles. Moreover, the possibility of function-specific modulation, such as selectively targeting p97’s roles in ERAD, CAD, or aggrephagy, opens new avenues for precision therapeutic approaches. Combination therapies represent another promising strategy. There is a clear preclinical rationale supporting the combination of p97 inhibitors with proteasome inhibitors, autophagy disruptors, DNA-damaging agents, HDAC inhibitors, and immune checkpoint inhibitors. These rational combinations can synergistically trigger apoptosis-inducing levels of cellular stress, overcome mechanisms of drug resistance, and broaden the therapeutic index of p97 inhibitors. While novel combination therapies may enhance the anticancer efficacy of p97 inhibitors, care must be taken as this may also result in additional adverse events.

Moving forward, future research must prioritize the identification of biomarkers of p97 dependency to enable patient stratification and precision therapy. A deeper understanding of tumor-specific reliance on p97’s distinct functions will be essential for guiding clinical decision-making. In parallel, combinatorial regimens should be optimized to maximize efficacy while minimizing toxicity. Their clinical translation should ideally be supported by predictive preclinical models and translational biomarkers of response to optimize success.

## 14. Conclusions

In summary, p97 represents a uniquely positioned therapeutic target at the interface of protein homeostasis, genome stability, and transcriptional control. Continued investment in drug discovery, mechanistic insight, and rational combination strategies holds the promise of transforming p97 inhibition from a conceptual opportunity into a clinically impactful cancer therapy.

## Figures and Tables

**Table 1:** Role of p97 in selected solid malignancies.

Solid Tumor	p97/VCP Role	Key Findings	Refs
Colorectal Cancer	Overexpressed; linked to higher invasion, metastasis, and poor prognosis	High expression correlates with deeper invasion, advanced stage, and recurrence	[7]
Bladder Cancer	Elevated in muscle-invasive forms	Interacts with the MRN complex and DNA damage repair	[7]
Breast Cancer	Highly expressed in cancer stem-like cells	Promotes cancer stem cell phenotype partly via unfolded protein response; inhibition induces paraptosis selectively in cancer cells	[8]
Non-Small Cell Lung Carcinoma (NSCLC)	Frequently upregulated; associated with tumor growth and migration	Inhibition by siRNA/small molecules suppresses proliferation, migration, and induces apoptosis	[57]
Squamous Cell Carcinoma	Essential for cancer cell survival	Knockdown induces selective cell death in cancer cells through ER and amino acid stress responses	[58]
Pancreatic Cancer	Facilitates migration and invasion	p97 regulates invasion/migration of pancreatic cancer cells	[59]

**Table 2:** High expression of p97 in various cancer types.

Cancer Type	Clinical Outcome	Refs
Colorectal Cancer	Increased invasion, higher recurrence rate	[86]
Pancreatic Ductal Adenocarcinoma	Poor survival outcomes	[59]
Non-Small Cell Lung Cancer	Enhanced metastatic potential, chemoresistance	[57]
Multiple Myeloma	Poor prognosis, increased tumor aggression	[87]
Melanoma	Increased tumor progression	[88,89]
Breast Carcinoma	Poor survival, higher recurrence	[8]
Hepatocellular Carcinoma	Increased tumor growth, poor prognosis	[90]
Ovarian Cancer	Chemoresistance, poor survival	[91,92]

**Table 3:** Characteristics of p97 Inhibitors.

Inhibitor	Mechanism of Action	Preclinical Outcome
CB-5083	ATP-competitive, D2 domain	Broad efficacy in multiple myeloma, colorectal, and pancreatic cancer models
CB-5339	ATP-competitive, D2 domain, enhanced potency	Activity in glioblastoma, lymphoma, ovarian cancer, and AML models
DBeQ	ATP-competitive, D1 and D2 domains	Induces caspase-dependent apoptosis in leukemia and solid tumor models
ML240	ATP-competitive, D2 domain	Anti-tumor effects in leukemia and solid tumors
ML241	ATP-competitive, D2 domain	Moderate efficacy in tumor models
NMS-873	Allosteric inhibitor with high selectivity	Potent activity in both solid and hematologic malignancies
UPCDC-30245	Allosteric, disrupts cofactor interactions	Inhibits lysosomal degradation; effective in colorectal cancer models
Eeyarestatin I	Indirect inhibitor; impairs ERAD-associated DUBs	Induces ER stress and cytotoxicity in leukemia and multiple myeloma cells

**Table 4:** Clinical trials for CB-5083 and CB-5339.

Inhibitor	Trial ID	Cancer Type	Patients (n)	Phase
CB-5083	NCT02243917	Advanced solid tumors	62	Phase 1
CB-5083	NCT02223598	Lymphoid hematological malignancies	120	Phase 1
CB-5339	NCT04372641	Advanced solid tumors and lymphoma	Not specified	Phase 1
CB-5339	NCT04402541	Acute myeloid leukemia and high-risk/MDS	55	Phase 1

## Data Availability

As this is a review article, no new data were created. Data availability is not applicable.

## Abbreviations

The following abbreviations are used in this manuscript:

AAA	ATPases Associated with diverse cellular activities
ATF6	Activating Transcription Factor 6
BER	Base Excision Repair
CAD	Chromatin-Associated Degradation
CHOP	C/EBP Homologous Protein
DDR	DNA Damage Response
DLT	Dose-Limiting Toxicity
DSB	Double-Strand Break
DUB	Deubiquitinating Enzyme
ER	Endoplasmic Reticulum
ERAD	Endoplasmic Reticulum-Associated Degradation
HCQ	Hydroxychloroquine
HDAC	Histone Deacetylase
HRR	Homologous Recombination Repair
ICL	Interstrand Crosslink
IRE-1α	Inositol-Requiring Enzyme 1α
LC3	Microtubule-Associated Protein 1 Light Chain 3
MM	Multiple Myeloma
MMR	Mismatch Repair
NER	Nucleotide Excision Repair
NHEJ	Non-Homologous End Joining
NSCLC	Non-Small Cell Lung Cancer
PARP	Poly(ADP-ribose) Polymerase
PDAC	Pancreatic Ductal Adenocarcinoma
PERK	PKR-like ER Kinase
PFS	Progression-Free Survival
PICHROS	Protein-Induced Chromatin Stress
PTM	Post-Translational Modification
THBS1	Thrombospondin-1
UPR	Unfolded Protein Response
UPS	Ubiquitin-Proteasome System
VCP	Valosin-Containing Protein
